# Toward Personalized Heart Failure Management: Integrating Biomarkers and Multimodal Monitoring Strategies

**DOI:** 10.1007/s11897-026-00742-3

**Published:** 2026-03-04

**Authors:** Youssra Allach, Mylène Barry - Loncq de Jong, Jasper J. Brugts

**Affiliations:** Department of Cardiology, Erasmus MC, University Medical Center Rotterdam, Rotterdam, The Netherlands

**Keywords:** Biomarkers, Biomarker-guided therapy, Repeated measurements, Risk stratification, Pathophysiology

## Abstract

**Purpose of Review:**

Effective heart failure (HF) management requires timely recognition and treatment of congestion in both inpatient and outpatient settings. As the complexity of HF care increases, there is a growing interest in improved guided therapy strategies, including both biomarker-driven approaches and non-invasive and invasive remote monitoring.

**Recent Findings:**

While natriuretic peptides remain the cornerstone of biomarker-based prognostication, emerging biomarkers reflecting diverse pathophysiological processes, such as myocardial stress, fibrosis, and inflammation, offer potential for enhanced risk stratification, particularly when used in serial multimarker panels. Despite their prognostic value, biomarkers are not yet routinely employed in guided therapy. Additionally with the increasing availability hemodynamic remote monitoring strategies and devices, we could learn more about pathophysiological processes, multi-biomarker panels and their relation to congestion and risks for progression of HF which can help to further refine biomarker-guided therapy.

**Summary:**

This review evaluates the potential use of biomarkers in guided therapy for HF and explores the rationale for their integration into multimodal monitoring frameworks aiming for a more personalized and dynamic model of HF care, in which different monitoring approaches can reinforce each other.

## Introduction

**Fig. 1:**
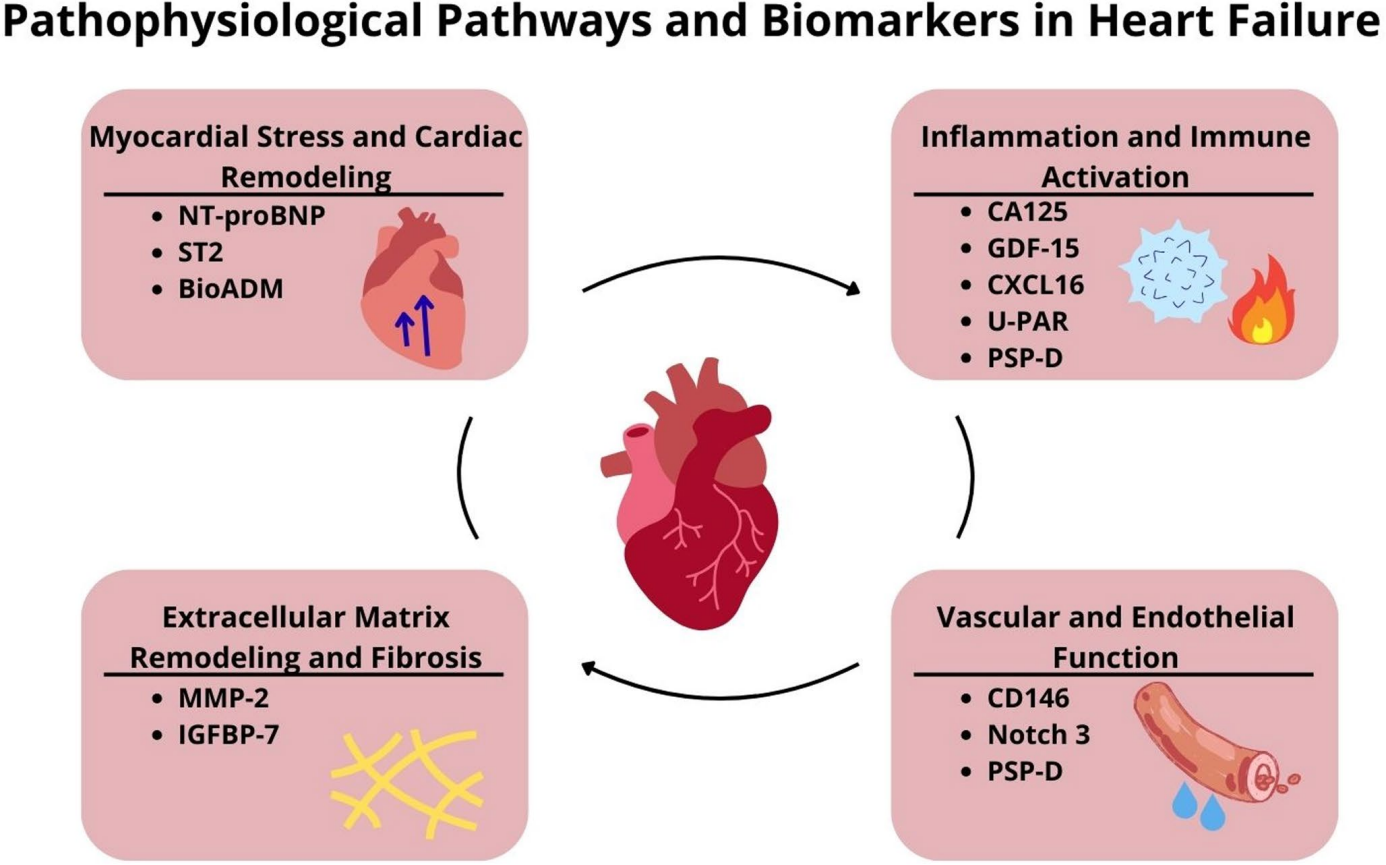
Pathophysiological pathways and biomarkers in heart failure

The complexity of heart failure (HF) pathophysiology calls for a shift from standardized therapy approaches to more personalized management strategies that account for individual disease trajectories [1, 2]. In recent years, biomarker-guided therapy has been proposed as a promising strategy for improving HF management [3–5]. Biomarkers provide valuable insights into pathophysiological processes, including myocardial stress, fibrosis, inflammation, and fluid overload (Fig. 1). Moreover, circulating biomarkers play an important role in prognostication [6, 7]. While natriuretic peptides, such as NT-proBNP, are well established in clinical practice for diagnosing and predicting HF outcomes, therapy guided solely by NT-proBNP yielded inconsistent results [8]. However, recent advances in multimodal monitoring, integrating biomarkers with hemodynamic data, have emerged as potentially novel strategies to enhance HF management and address the limitations of traditional previous approaches [9].

Recent studies suggest that integrating biomarker panels with multiple biomarkers enhances both sensitivity and specificity for HF prognostication [10]. Moreover, repeated measurement of biomarkers has been shown to provide a more reliable and accurate assessment of pathophysiological processes and prognosis in HF patients [11]. While biomarkers alone have shown promise, their combination with real-time telemonitoring modalities has the potential to enhance pathophysiological understanding and early detection of decompensation and guide timely interventions. In the future, this approach could possibly enable a more comprehensive assessment of changes in biological characteristics through biomarker profiling [12].In recent years, there have been advances in the availability of invasive and non-invasive remote monitor sensors related to hemodynamics. The hemodynamic data revealed by these techniques could be combined with blood-biomarkers to gain more insight in their patterns and pathophysiological relationships with congestion to refine and improve biomarker-guided therapy strategies [13].

Blood-biomarkers remain relatively underutilized in the context of remote cardiac monitoring strategies for patients with HF, especially when compared to widely adopted non-invasive modalities such as various telemonitoring approaches [13]. A more comprehensive understanding of the interplay between circulating biomarker profiles and hemodynamic parameters could pave the way for more precise and individualized therapeutic interventions, ultimately enhancing broader patient outcomes in HF management.

This review provides an overview of the role of biomarkers in the diagnosis, risk stratification and management of HF, with a focus on two emerging themes.

To this end, we explore various monitoring strategies. We first discuss the requirements for the ideal blood biomarker, and which biomarkers are already known in the context of congestion in heart failure, and what insights biomarker-guided therapy studies have provided us so far. We then discuss recent insights into non-invasive and invasive monitoring strategies of heart failure patients that could enrich the field of biomarker guided therapy. Finally, we will discuss future perspectives, in which we will explore how the integration of biomarker data with remote monitoring measurements, including the application of Artificial Intelligence (AI) and implantable sensors, may offer new opportunities to improve pathophysiological insights, disease monitoring and therapeutic decision-making.

## Criteria for Clinically Useful Biomarkers in Heart Failure

Biomarkers play an important role in the diagnosis, risk stratification and management of HF. However, their clinical utility depends on several criteria. An ideal biomarker must demonstrate biological validity, with a clear link to HF pathophysiology, and must be reproducible across various settings [8]. It should also provide prognostic and therapeutic value offering reliable insights into disease progression and support clinical decision-making. Additionally, biomarkers must be clinical applicable and interpretable, easy to measure, and cost-effective for routine use.

### Biological Rationality

Biomarker linked to critical processes such as myocardial stress, myocardial remodeling, inflammation and congestion provide valuable insights into disease severity and progression [14, 15]. For instance, biomarkers like NT-proBNP and ST2, which are associated with myocardial stress and congestion provide meaningful prognostic value. When combined with fluid status or hemodynamic parameters, such as weight, blood pressure, and other signs of fluid status, these biomarkers enable real-time disease monitoring and dynamic tracking of disease progression. This integration is important for both diagnostic and therapeutic guidance, offering a more holistic understanding of HF.

### Reproducibility and Reliability

For a biomarker to be integrated into clinical practice, it must provide consistent and reliable results across various settings and patient populations [14, 15]. Variability caused by comorbidities, physiological changes, or differences in assays can weaken its clinical utility.

### Prognostic and Therapeutic Value

Within the context of congestion and clinical deterioration in HF, biomarkers should offer valuable prognostic insights and help guide therapeutic decisions [14, 15]. Biomarkers related to congestion (e.g., ST2, CA125, bio-ADM) have been shown to predict clinical outcomes and guide treatment decisions by indicating the severity of fluid overload and cardiac dysfunction.

### Clinical Applicability and Timing in the Disease Process

Even if a biomarker demonstrates biological validity, reliability, and prognostic value, its widespread use depends on its feasibility for routine clinical practice [8, 15]. A clinically applicable biomarker must be easy to measure, cost-effective, and accessible in different healthcare settings.

**Fig. 2:**
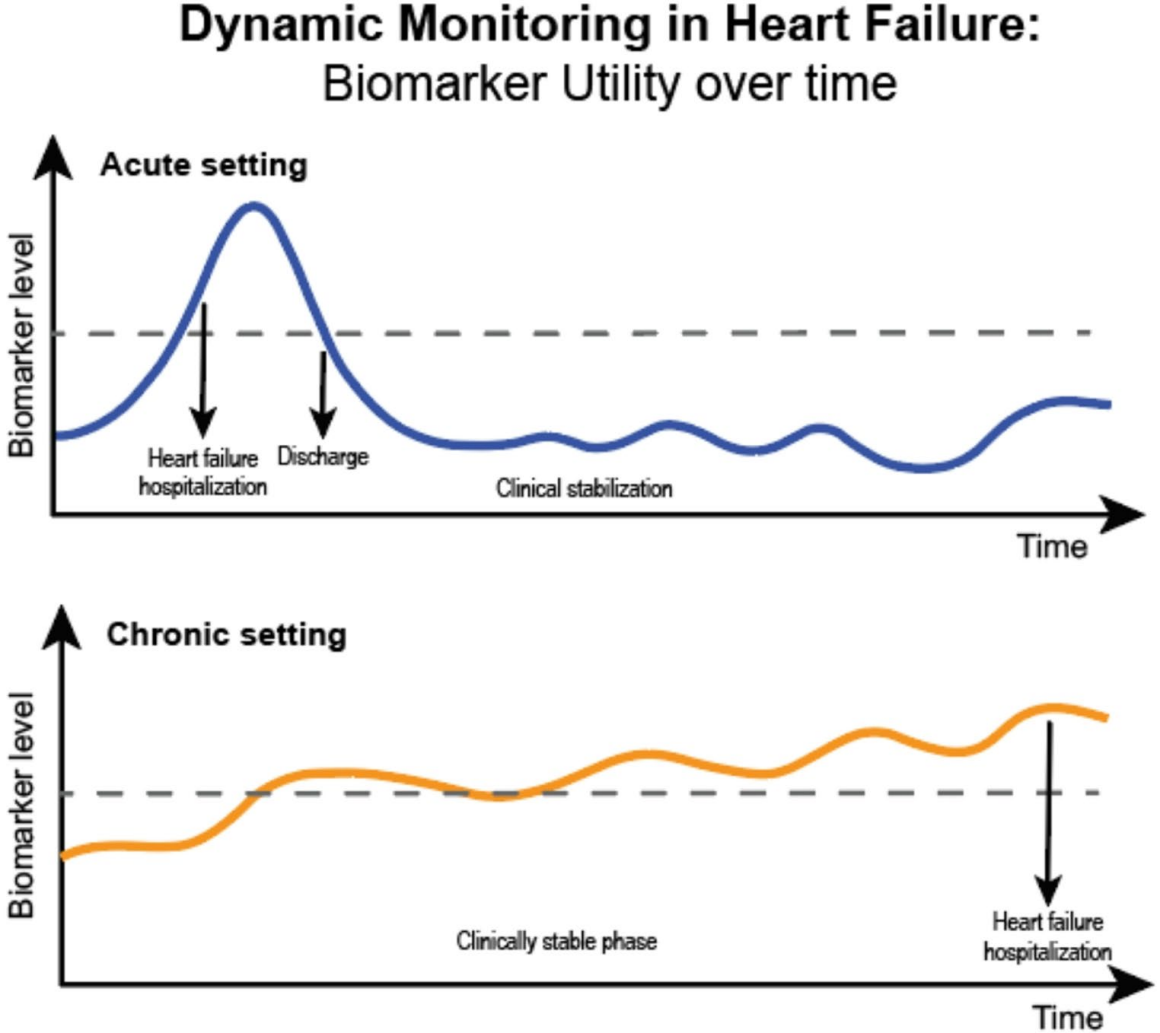
The dynamic change of biomarker values over time. Legend: The blue line indicates the course of clinically useful biomarkers, such as NT-proBNP, in the acute setting. The orange line indicates this biomarker-level course in a chronic setting. The dashed line indicates the upper limit of the biomarker’s normal value. In the acute setting, there is a rapid clinical deterioration of HF accompanied by a rapid rise in biomarker level. After timely initiation of therapy, this biomarker reaches a maximum, after which it drops again and stabilizes. In a chronic setting, both the patient’s clinical features and biomarker values remain stable for an extended period of time, although biomarker levels may rise slowly leading up to a clinical event

An important factor in this regard is the timing of biomarker application throughout the disease process. In the acute phase, biomarkers such as NT-proBNP rise rapidly during clinical deterioration (Fig. 2) [16]. After initiation of therapy, biomarkers peak and then decline. In chronic HF, biomarkers typically remain stable or rise slightly over time, until a clinical event occurs [17]. Established biomarkers, such as natriuretic peptides, are the standard for clinical use, while emerging markers are still being evaluated [7].

A significant disadvantage of multiple prognostically relevant congestion biomarkers is that they are not yet clinically applicable. In the future, broader clinical availability of these markers would offer the possibility of developing a prognostically relevant biomarker panel. It should be integrated into standardized care pathways to become a regular part of patient management. Ideally, one or more biomarkers could be measured in a very accessible manner, such as via a point-of-care method in the home setting [18–20]. This would make biomarker-driven care more practical, accessible, and faster to implement.

## Biomarkers and their Clinical Relevance in Heart Failure

Congestion-related biomarkers are particularly important in HF prognostication and disease management, as fluid overload is a driving factor in HF progression and decompensation. Traditional markers such as NT-proBNP reflect myocardial stretch and volume overload, while newer biomarkers offer insights into processes such as inflammation, endothelial dysfunction, and cardiac and extracellular matrix remodeling (Fig. 1) [6, 7]. Moreover, several biomarkers are known to be associated with pulmonary artery pressures and clinical congestion [6, 9]. In the following sections, both conventional and newer emerging biomarkers are discussed, with a focus on their prognostic significance in HF.

### Biomarkers of Myocardial Stress and Cardiac Remodeling

Myocardial stress results from increased wall tension due to pressure or volume overload, while cardiac remodeling involves structural and functional changes, including fibrosis and ventricular dilation. Among the biomarkers that reflect these processes, NT-proBNP and ST2 are the most established [6].

 NT-proBNP is released in response to ventricular wall stress linked to elevated filling pressures. Increased levels of NT-proBNP correlate with worsening congestion symptoms, increased risk of hospitalization and mortality, making it an useful biomarker for assessing volume status and guiding decongestive therapy [23].

 ST2, a member of the interleukin-1 receptor family, is mainly associated with cardiac remodeling, fibrosis and inflammatory processes [6, 24]. Elevated levels of ST2 have been correlated with poorer outcomes in HF, and when combined with NT-proBNP, they enhance prognostic accuracy.

 BioADM, an active form of adrenomedullin, plays an important role in regulating vascular tone and maintaining endothelial function. In HF, elevated Bio-ADM levels correlate with clinical congestion and hemodynamic stress, such as increased pulmonary capillary wedge pressure. It has shown potential as a biomarker for assessing congestion and guiding therapy, particularly in diuretic treatment [6, 25–29]. However, further research is needed to clarify its long-term value in monitoring congestion and influencing clinical outcomes.

### Biomarkers of Extracellular Matrix (ECM) Remodeling and Fibrosis

ECM remodeling and fibrosis are important processes in HF, as they contribute to impaired cardiac function and exacerbate fluid accumulation, leading to congestion [24, 30]. ECM remodeling involves changes in the composition of the ECM, which can affect the heart’s structure and its ability to function properly. Fibrosis is the excessive buildup of fibrous tissue, which can impair normal heart function and lead to higher filling pressures. In this context, we explore three important biomarkers: MMP-2 and IGFBP-7.

 MMP-2 (Matrix metalloproteinase-2) is essential for breaking down the ECM and supporting the remodeling process [31]. Higher levels of MMP-2 have been linked to the development of myocardial fibrosis, which can lead to stiffness in the heart and difficulty with filling during the diastolic phase. Increased MMP-2 activity is associated with worse outcomes in HF patients [32, 33].

 IGFBP-7 (Insulin-like growth factor-binding protein 7) plays a role in regulating insulin-like growth factors, which are important for cell growth and survival [24]. In HF, IGFBP-7 is associated with fibrotic changes in the heart muscle. Increased levels of IGFBP-7 have been linked to increased cardiac fibrosis, a higher risk of incidence HF and poorer outcomes in HF patients [10, 34, 35].

### Biomarkers of Inflammation and Immune Activation

Inflammation and activation of the immune system play an important pathophysiological role in the progression of HF. An exaggerated inflammatory response can lead to adverse cardiac changes and exacerbate fluid overload. Monitoring specific biomarkers associated with inflammation and immune activation could provide valuable insights into these processes and aid in assessing the severity of congestion in HF. In this context, CA125, GDF-15, CXCL16, U-PAR, and PSP-D are discussed in this section.

 CA125 (mucin 16), is a glycoprotein primarily associated with volume overload conditions, including HF. Elevated CA125 levels correlate with tissue congestion, increased cardiac filling pressures, and right-sided HF, particularly in the presence of pleural effusion and peripheral edema [36]. Moreover, CA125 levels are significantly associated with HF disease severity, and its trajectory over time can predict long-term mortality and readmission in HF patients. CA125 has been suggested as a tool for guiding diuretic therapy, with studies showing that CA125-guided adjustments in diuretic treatment can improve outcomes. The biomarker is not influenced by age, kidney function, or left ventricular status, making it valuable for assessing congestion in a variety of HF phenotypes, including HFpEF [6, 36–40].

 GDF-15 (growth differentiation factor 15) is a cytokine linked to stress and inflammation [41]. Elevated levels of GDF-15 are associated with worsening cardiac function and increased filling pressures.

Upcoming biomarkers like CXCL16, U-PAR, and PSP-D are linked to congestion-related processes, such as inflammation and vascular dysfunction. These could offer a better understanding of HF pathophysiology and management of fluid overload in HF. CXCL16 is a chemokine involved in immune cell recruitment to areas of inflammation. Elevated CXCL16 levels in HF are associated with increased inflammation, myocardial injury, and worse clinical outcomes, highlighting its potential role in fluid overload and broader vascular inflammation [42–44]. U-PAR (urokinase-type plasminogen activator receptor) regulates immune responses and tissue remodeling, with elevated levels in HF linked to increased inflammation, fibrosis, and fluid retention [45]. PSP-D (pulmonary surfactant-associated protein D) plays a role in regulating immune responses and maintaining vascular homeostasis. Elevated PSP-D levels in HF patients are associated with inflammation, endothelial dysfunction, and hemodynamic compromise, suggesting its potential as a marker for both inflammation and fluid overload [46–48].

### Biomarkers of Vascular Function and Endothelial Dysfunction

Vascular function and the health of the endothelium are important aspects in understanding HF [6]. Endothelium dysfunction can lead to issues such as reduced blood vessel dilation, increased permeability of the blood vessel, and inflammation, worsening the overall hemodynamic status of patients with HF. In this context, lesser known biomarkers like (s)CD146, Notch 3 and PSP-D are recognized for their potential roles in endothelial dysfunction and vascular remodeling. These biomarkers could be of interest because they may provide additional insight into the pathophysiological processes that drive vascular alterations in HF. In this section, we will explore three important biomarkers: (s)CD146, Notch 3, and PSP-D and their roles in vascular function and endothelial dysfunction within the context of HF.

 CD146, is a glycoprotein, of which the soluble form of CD146 (sCD146) is overexpressed in conditions involving inflammation, vascular injury, and endothelial dysfunction,. Elevated sCD146 levels have been associated with peripheral edema and venous congestion, suggesting its potential as a biomarker for congestion in HF. Although the evidence supporting its clinical utility is limited, preliminary studies indicate that sCD146 could help differentiate between central and peripheral congestion [6, 49, 50].

 Notch 3 (neurogenic locus notch homologue protein 3) plays an important role in endothelial cell signaling and vascular remodeling, both essential for maintaining vascular homeostasis [51]. Dysregulation of Notch 3 signaling has been implicated in various cardiovascular diseases, including HF, where it may contribute to endothelial dysfunction and adverse vascular remodeling. While studies linking Notch 3 to HF are scarce, alterations in Notch 3 levels appear to correlate with the severity of HF, suggesting its potential as a biomarker for monitoring vascular health and congestion [52].

 PSP-D, as discussed in Section II.c, also modulates immune responses affecting endothelial function [45].

Although many of the biomarkers discussed above show promise in explaining pathophysiological processes, predicting clinical deterioration and clinical events and possibly also in assisting disease management in heart failure patients, most of these markers are not yet available or regularly used in clinical practice. Further research is still needed to fully validate their clinical usefulness in monitoring congestion and tailoring therapy. In addition, research into integration with other monitoring modalities is useful, since different monitoring strategies may reinforce each other in the context of congestion at HF.

## Biomarker-Guided Therapy in Acute vs. Chronic Heart Failure: Current Insights and Lessons Learned

HF can present as either acute heart failure (AHF) or chronic heart failure (CHF). Both require different management strategies. The use of biomarkers in prognostication and therapy has become an important tool for optimizing care, by enabling early diagnosis, risk stratification, and treatment monitoring [53]. However, the clinical application of biomarkers differs between these patient groups due to their pathophysiological characteristics and treatment goals [54]. In AHF, biomarkers could support rapid decision-making, guiding interventions at admission and discharge [55]. In CHF, serial biomarker measurements allow for long-term monitoring, helping to assess disease progression and adjust therapy accordingly [4]. In this section, we discuss the use of biomarkers in different types of HF and what we can learn about this from previous studies.

### Biomarker-Guided Therapy in Acute and Chronic Heart Failure

The use of biomarkers for risk stratification and guiding interventions differs between AHF and CHF. AHF is characterized by rapid onset of or deterioration of symptoms, often with clinical decompensation due to congestion, volume overload or myocardial injury [6]. The primary aim in managing AHF is to achieve clinical stabilization and prevent deterioration or hospitalization. In this context, biomarker assessment helps clinicians to quickly identify high-risk patients, guide intensive inpatient management, and structure follow-up post-discharge [56]. In CHF, the main focus is on long-term disease monitoring and therapy optimization [54]. Unlike AHF, where biomarker assessment is primarily used during hospitalization, CHF management benefits from serial biomarker measurements that provide insights into disease progression and treatment response over time [10, 11, 47]. This allows healthcare providers to detect subtle changes that may indicate early decompensation, possibly enabling early intervention and preventing clinical decline.

Natriuretic peptides such as B-type natriuretic peptide (BNP) and NT-proBNP are well-established biomarkers in both AHF and CHF [6, 7]. Persisting elevated NT-proBNP levels at discharge from hospitalization for decompensated HF are associated with a higher risk of early readmission and mortality [57]. Serial measurements during hospitalization allow for treatment monitoring, guiding decisions on diuretics, vasodilators, and inotropic therapy. In CHF management, trends in NT-proBNP levels serve as indicators of therapy effectiveness or disease progression [5, 58–60]. A decline in NT-proBNP levels reflects effective treatment and stabilization of HF, while persistent elevation of NT-proBNP levels, despite optimized treatment, may indicate insufficient decongestion, ongoing myocardial stress or comorbidities complicating management.

Although well-established as prognostic markers, the additional benefit of a biomarker-guided treatment strategy based on natriuretic peptides has not been conclusively demonstrated in clinical practice. While natriuretic peptide-guided therapy has shown promise, clinical trials such as GUIDE-IT, PRIMA II, PROTECT, TIME-CHF and BATTLE-SCARRED have yielded mixed results (Table 1) [61–63]. GUIDE-IT found that NT-proBNP-guided therapy in HFrEF did not significantly improve outcomes compared to standard care, despite achieving greater NT-proBNP decrease [61]. Similarly, PRIMA II showed no significant benefit in mortality or readmissions for patients with acute decompensated HF, although more patients in the biomarker-guided group achieved a ≥ 30% NT-proBNP decline [62]. The PROTECT trial demonstrated that NT-proBNP-guided therapy could lead to better volume management and a reduction in cardiovascular events [63]. The TIME-CHF trial, conducted in older patients with chronic HF, showed that NT-proBNP-guided therapy improved outcomes in patients younger than 75 years, but not in the older subgroup, implying the impact of age on treatment effect [64]. Likewise, the BATTLE-SCARRED study found that biomarker-guided therapy was associated with reduced long-term mortality in patients under 75 years of age, but not in the overall cohort [65]. Moreover, several meta-analyses, which also included several smaller cohorts in addition to the studies mentioned above, have yielded conflicting or inconclusive evidence regarding natriuretic peptide-guided therapy in HF, although differences appear to exist across age groups and ejection fraction categories [4, 55].

**Table 1:** Overview of randomized clinical trials on NT-proBNP guided therapy in heart failure

Trial	Population	Sample size (*n*)	Follow-up duration	Intervention	Comparator	Primary outcome	Results	Effect size, HR Ratio	*P* Value	Subgroup analysis	Conclusions
BATTLE-SCARRED (2006)	CHF, < 75 years	364	3 Years	NT-proBNP-guided therapy	Clinical care	3-year mortality	Reduced mortality in patients < 75 years	HR 0.75	*P* = 0.02	Younger patients, no benefit in > 75 years	Benefit mainly in younger patients, limited generalizability
PROTECT (2008)	CHF, outpatient setting	1110	2 Years	NT-proBNP ≤ 1000 pg/ml	Standard of care	HF events, EF, ventricular size	Fewer HF events, improved EF and ventricular size	HR 0.80	*P* = 0.04	Subgroup with lower baseline EF showed better response	Positive effects, selective population, relatively small study
TIME-CHF (2008)	CHF, age groups < 75 vs. ≥ 75	1003	3 Years	NT-proBNP-guided therapy	Clinical care	Composite mortality and hospitalizations	No overall benefit, better results in < 75 years subgroup	HR 0.85* * For < 75 years	*P* = 0.25 (Overall)	Better results in patients < 75 years	Age modifies effect, no overall benefit
PRIMA II (2018)	Acute decompensated HF, HFrEF	1234	6 Months	≥ 30% NT-proBNP reduction during admission	Standard of care	Mortality and HF re-admissions within 6 months	No significant difference in primary outcome, but more patients achieved ≥ 30% NT-proBNP reduction	HR 0.98	*P* = 0.56	No effect in subgroups	Biomarker reduction does not translate into clinical benefit in acute setting
GUIDE-IT (2018)	Chronic HFrEF, high-risk	1600	3 Years	NT-proBNP < 1000 pg/ml	Standard of care	CV death or HF hospitalization	No significant difference in primary outcome; greater NT-proBNP reduction in intervention group	HR 0.98	*P* = 0.88	Better NT-proBNP reduction but no benefit in outcomes	Mixed results; guided therapy did not improve hard outcomes despite NT-proBNP reduction

Although the concept of biomarker-guided therapy appears promising, current evidence does not sufficiently support the routine measurement of BNP or NT-proBNP for therapy titration to warrant formal recommendation in clinical guidelines [54]. This implies that there may be a gap in knowledge regarding the application and approach of biomarker-guided therapy, particularly the overreliance on single biomarkers, to guide treatment decisions. Biomarkers like NT-proBNP have proven useful in assessing cardiac stress and fluid overload, as indicators of prognosis, but fail to capture the full complexity of HF pathophysiology. In the future, a congestion panel could potentially be an appropriate solution to this challenge. In this context, the composition of a multi-biomarker panel, including for example ST2, BioADM, and CA125, could provide a more comprehensive view of underlying pathophysiological mechanisms that contribute to the development of congestion and clinical deterioration [6]. Future research should focus on integrating these biomarkers into a comprehensive framework that provides better insight into HF progression. A summary of advantages and disadvantages of a multimarker approach compared with a single biomarker measurement is shown in Table 2.

**Table 2:** Advantages and disadvantages of a multimarker approach compared to a single biomarker measurement

	Single biomarker measurement	Multi-biomarker measurements
Pros	Simple interpretation and integration into clinical workflow. Established evidence (e.g. NT-proBNP and BNP are well-validated for diagnosis and prognostication). Lower laboratory and resource costs.	Captures diverse HF pathophysiology (e.g. myocardial injury, fibrosis, inflammation, etc.) Improved prognostic accuracy and risk stratification.Potential for personalized care: tailored therapy based on individual risk profiles.
Cons	Limited pathophysiological insights. Affected by confounders (e.g., renal function, BMI), resulting in potentially missed risk stratification in case of other active pathological processes. Ceiling effect: potential prognostic power plateaus, especially in case of complex comorbidities.	Complex interpretation requiring advanced analytics and/or interpretation algorithms. Higher cost due to increased laboratory expenses and resources. Potential redundancy: due to high correlation of multiple biomarkers, the added effect might be small. Unclear clinical utility: no universal or widely applicable multi-marker strategy is available for clinical use yet.

In addition to traditional outcomes such as hospitalization and mortality, broader endpoints such as hemodynamic parameters, symptom burden or quality of life could be explored. These outcomes could provide clinicians with a more nuanced view of a patient’s condition. Furthermore, it is important that relevant biomarkers become clinically available and can be easily obtained, for example in a point-of-care or hospital-at-home situation. In addition, it is likely that biomarker-based guidance approaches can be enhanced by integrating other measurements and modalities, including remote monitoring strategies using AI, telemonitoring, or invasive measurements. This warrants further investigation in the future. By addressing the gaps in the current framework, risk stratification and the initiation of timely interventions could be improved, thus improving HF management and patient outcomes.

### Biomarker Guided Therapy in HF with Reduced and Preserved Ejection Fraction (HFrEF vs. HFpEF)

Distinct biomarkers have been associated with prognosis and clinical deterioration in heart failure with reduced ejection fraction (HFrEF) compared to heart failure with preserved ejection fraction (HFpEF). These differences must be considered when selecting biomarkers for studies aimed at developing biomarker-guided therapy strategies. Tailoring biomarker selection to the specific HF phenotype may improve the precision and effectiveness of such approaches. In HFrEF, biomarkers like NT-proBNP and troponins are more reliable indicators of cardiac stress and myocyte injury compared to HFpEF [7]. In contrast, pathophysiology in HFpEF is often heterogeneous, and can be influenced by comorbidities such as chronic kidney disease, obesity, inflammatory diseases, and atrial fibrillation, which complicates biomarker interpretation [41, 66]. Natriuretic peptides, which are highly reliable in HFrEF, have less predictive value in HFpEF due to factors like obesity and renal impairment. Emerging biomarkers linked to inflammation and ECM remodeling, such as GDF-15 and galectin-3, may offer new insights into HFpEF pathophysiology and prognostication. Studies suggest that GDF-15 is associated with increased risk of incident HFpEF, reflecting pathways of inflammation [41].

A multimarker approach, combining biomarkers, may enhance risk stratification and therapeutic decision-making especially in HFpEF, since it could account for the complexity of this syndrome.

## Non-invasive Monitoring Techniques and their Additive Role To Biomarkers in Prognostication and Guided Therapy

As the role of biomarkers in monitoring HF progression has grown. Telemonitoring modalities, which track patient data remotely, offer a complementary approach from a different angle. We believe that integrating these two strategies could enhance patient care and management in HF. Non-invasive monitoring techniques have become important in HF management, enabling continuous tracking of patient status without the need for invasive procedures with associated risks. These technologies, including wearable devices, sensor-based systems, and telemonitoring platforms allow clinicians to monitor physiological parameters in real-time [67, 68].

Recent research, including meta-analyses highlight the growing promise of non-invasive monitoring systems. Wearable devices, such as ECG patches and blood pressure monitors, enable real-time tracking of parameters like heart rate, ECG, and oxygen saturation in HF patients. This continuous monitoring allows early detection of worsening symptoms and facilitates timely, data-driven interventions. However, proof for a general positive effect on clinical outcomes is still limited due to the heterogeneity among applied modalities and intervention strategies [67].

AI-driven algorithms are becoming increasingly important in optimizing noninvasive monitoring approaches. These algorithms could process large datasets from wearable devices, biomarkers, and telemonitoring platforms to facilitate automated risk stratification and early detection of decompensation [68, 69]. Research is ongoing to refine these models, aiming to improve their predictive accuracy and integrate them seamlessly into clinical practice. As AI systems become more reliable, they hold great potential to enhance the understanding of HF as well as to personalize treatment by providing real-time, actionable insights that guide clinicians in making informed decisions In addition, studies have demonstrated that combinations of non-invasive measurements and biomarkers, based on AI-algorithms, can provide accurate prognoses [70]. Future research will have to show whether multimodal prognostic models, potentially developed with the assistance of AI, can indeed improve the prognosis and management of HF patients.

## Invasive Monitoring Techniques in Heart Failure

Invasive hemodynamic monitoring plays an increasing role in the management of HF. These technologies require the implantation of sensors to directly measure hemodynamic variables such as pulmonary artery pressure (PAP), and left atrial pressure (LAP), all of which are important for assessing the filling pressures and severity of congestion [71, 72].

PAP sensors are among the most widely used invasive management tools. Mean PAP is measured, to assess filling pressures as surrogate marker of congestion. PAP-guided therapy can reduce HF-related hospitalizations and improve patient quality of life by allowing early intervention [13, 72]. LAP sensors, which measure pressures within the left atrium, also show potential in optimizing HF therapy. Complementary technologies such as the IVC collapsibility index and diameter monitoring devices, provide insights into volume status, helping assess venous pressure and detect early signs of fluid overload [73, 74].

The integration of these invasive monitoring systems with biomarkers significantly enhances our understanding of HF pathophysiology as well as the applicability of biomarker guided therapy [72–75]. Serial biomarker measurements, such as NT-proBNP, PSP-D, IGFBP-7, MMP-2 and CD93, have strong correlations with invasive hemodynamic measures like PAP. These biomarkers can provide complementary insights into hemodynamic changes, even for patients without invasive sensors, highlighting the potential for noninvasive biomarker-guided therapy alongside invasive monitoring [9].

Another advancement is the integration of biomarkers with digital health technologies, including wearable devices and implantable sensors. A multiparametric telemonitoring system, for example, combines biomarkers like NT-proBNP with sensor data from a wearable device, enabling the prediction of clinical worsening with a sensitivity of approximately 70% and specificity of 89% [76–78]. This integration highlight how combining biomarkers with real-time hemodynamic data can improve predictive accuracy and decision-making in HF management.

Despite the promising advancements in invasive hemodynamic monitoring, challenges remain in the widespread adoption, including cost and the need for specialized training. Ongoing research is needed to refine these technologies and validate their efficacy across diverse HF populations. Currently, the hemodynamic data provides a novel data on the evolution of worsening heart failure and the response to therapy.

## Future Perspectives

### The Integration of Biomarkers and Non-invasive or Invasive Monitoring To Boost Knowledge of Pathophysiological Processes in Heart Failure

Biomarker insight can improve from remote monitoring data and vice versa, regarding pathophysiological insights and management strategies. HF is a dynamic condition, and continuous monitoring of both biochemical and hemodynamic changes could be beneficial for optimizing treatment. Traditional approaches often rely on single-point biomarker assessments, which provide valuable but static insights into disease severity. In this context, the adoption of a multiparametric approach, integrating various prognostic and guidance tools, as previously proposed, could be a valuable strategy to address the limitations of single-modality monitoring [2, 6].

Recent studies have demonstrated the benefits of integrating biomarkers with clinical parameters in HF, particularly for guiding early interventions [9]. The GUIDE-IT trial integrated NT-proBNP measurements with clinical data, showing improvements in prognostic accuracy. Although the primary outcome of hospitalization and mortality did not significantly improve, the trial underscored the potential of this combined approach to enhance personalized risk assessments [61].

**Fig. 3:**
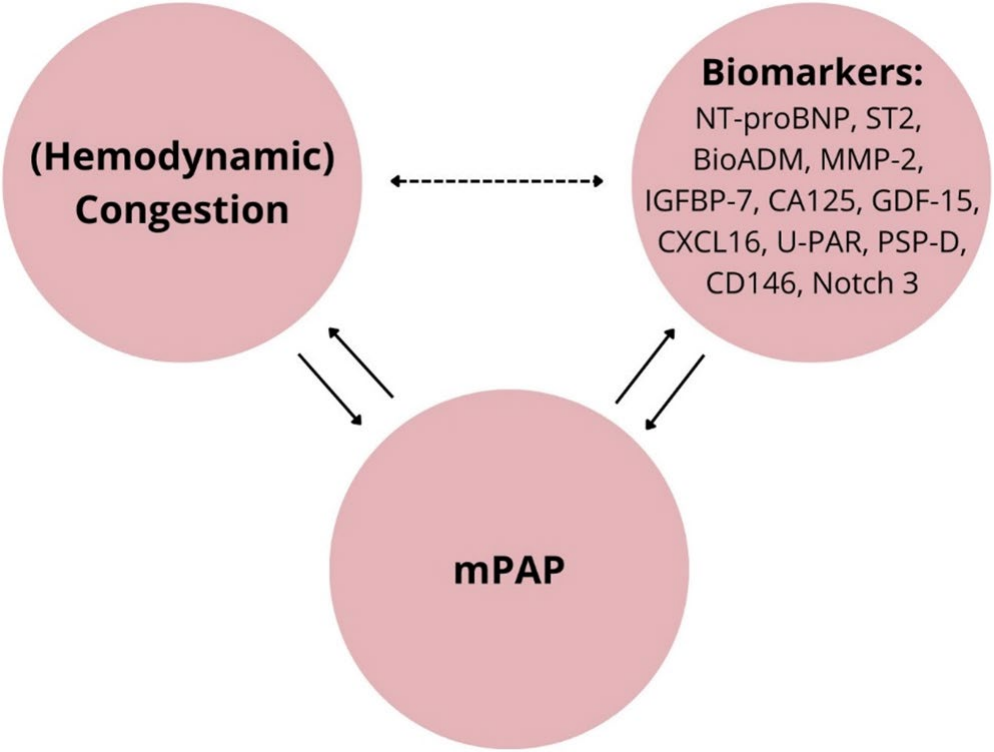
Integrated monitoring. An integrated approach to monitoring heart failure patients could provide a more comprehensive pathophysiological understanding of disease stage and risk factors

In particular, biomarkers, such as NT-proBNP, PSP-D, IGFBP-7, and MMP-2, have demonstrated strong correlations with hemodynamic parameters, suggesting their potential as early indicators of congestion and other pathophysiological processes such as myocardial stress, fibrosis and inflammation [6, 7, 9, 14, 47]. These biomarkers can be used to complement traditional hemodynamic monitoring, providing a more comprehensive understanding of the disease progression (Fig. 3). Unlike conventional threshold based strategies, monitoring biomarker trends over time helps distinguish between temporary fluctuations and true disease progression, supporting more personalized treatment strategies [10].

Furthermore, the potential role of point-of-care biomarker testing is becoming more evident [79]. This would allow patients to monitor relevant biomarkers in the home setting, increasing the feasibility and accessibility of long-term monitoring. Such a development could improve patient adherence to treatment plans, reduce hospital visits and ultimately enhance clinical outcomes by detecting changes earlier.

Despite these promising advancements, challenges remain in the standardization of both biomarker and mPAP-guided therapies. Future research should focus on refining biomarker algorithms, validating findings across diverse HF populations, and exploring non-invasive biomarker panels as complementary tools to hemodynamic monitoring. By integrating AI-driven algorithms with biomarker-guided therapy, researchers can refine risk stratification models and provide more precise insights into optimal treatment pathways for patients. Ultimately, combining serial biomarker measurements with clinical HF-parameters, obtained from noninvasive or invasive monitoring modalities, allows clinicians to gain a deeper understanding of disease progression and responses to therapy. Both fields could be enhanced and enriched by using and integrating the information provided bio markers or telemonitoring modalities.

### Integrating Biomarker Guided Therapy in a Multimodal Setting

Biomarker-guided therapy, as an easily accessible, minimally invasive management strategy, can benefit from emerging innovations in digital health, AI, and remote monitoring. These advancements have the potential to enable real-time disease monitoring, and improve early risk detection and personalized disease management. Further contributing to this is the recent development that testing biomarkers such as NT-proBNP at home via point-of-care analysis is becoming increasingly feasible, which lowers the threshold for biomarker analysis [19, 20]. The integration of AI-driven algorithms, home-based biomarker testing, and machine learning applications could enhance the clinical utility of biomarkers [80]. However, challenges related to cost, accessibility, and the need for standardized implementation must be addressed before these technologies can be widely adopted. Future research should focus on developing strategies for home-based testing of biomarkers or biomarker panels, such as serum creatinine or NT-proBNP, similar to the current glucose monitoring methods used in diabetes management. Just as continuous glucose monitoring has revolutionized diabetes care by allowing patients to manage their condition outside the traditional healthcare setting, a similar approach could be applied to HF biomarkers. This would enable patients to monitor their health at home, improving early detection and personalized treatment. Studies like the HABIT trial and the HOME HF study have already demonstrated the feasibility and clinical value of remote BNP monitoring for HF [19, 20]. These studies showed that daily home-based monitoring can track changes in biomarkers over time. Such an approach allows earlier intervention, potentially reducing hospital admissions and improving long-term patient outcomes. Furthermore, home-based biomarker monitoring could expand access to personalized HF management [18–20]. By combining these tests with smartphone apps, clinicians could make data-driven treatment adjustments based on biomarker trends [82], .

A biomarker-based approach in HF management offers several advantages. It is broader available, accessible, easily implemented in various clinical settings, and potentially more cost-effective than invasive monitoring methods. These benefits could be even further enhanced if point-of-care techniques for testing biomarkers, comparable to those used in glucose management for diabetics, were to become available for HF patients. However, limitations such as blood sampling discomfort, delays in obtaining results and within-person variability in biomarker levels must be considered. integrating biomarkers with invasive and non-invasive monitoring strategies could benefit HF pathophysiological understanding, prognostication and disease management, as information obtained by various techniques could complement each other. The integration of AI-driven models are becoming important in refining this multimodal approach. AI models can automate risk stratification, aiding the prediction of deterioration before clinical symptoms appear. Advances based on the integration of these multimodal management techniques, would and allow clinicians to make more informed decisions in real-time. Such advancements could ultimately improve patient outcomes, reduce hospital admissions, and enhance long-term management of heart failure. Ongoing research is necessary to validate these models and ensure their effectiveness across different patient groups, including those without access to remote monitoring.

## Conclusion

Future HF management could benefit and further refined from a more personalized and dynamic model that integrates serial biomarker measurements with remote monitoring modalities. The combination of these modalities potentially enhances early detection of congestion and disease progression, thereby supporting more timely and individualized therapeutic interventions. Given the central role of congestion in HF outcomes, its targeted assessment through multimodal monitoring congestion related biomarker strategies appears promising and could benefit broader populations.

## Data Availability

No datasets were generated or analysed during the current study.

## References

[1] Rosano GMC, Moura B, Metra M, Bohm M, Bauersachs J, Ben Gal T, et al. Patient profiling in heart failure for tailoring medical therapy. A consensus document of the heart failure association of the European society of cardiology. Eur J Heart Fail. 2021;23(6):872–81. 33932268 10.1002/ejhf.2206

[2] Girerd N, Seronde MF, Coiro S, Chouihed T, Bilbault P, Braun F, et al. Integrative assessment of congestion in heart failure throughout the patient journey. JACC Heart Fail. 2018;6(4):273–85. 29226815 10.1016/j.jchf.2017.09.023

[3] Felker GM, Hasselblad V, Hernandez AF, O’Connor CM. Biomarker-guided therapy in chronic heart failure: a meta-analysis of randomized controlled trials. Am Heart J. 2009;158(3):422–30. 19699866 10.1016/j.ahj.2009.06.018

[4] Savarese G, Trimarco B, Dellegrottaglie S, Prastaro M, Gambardella F, Rengo G, et al. Natriuretic peptide-guided therapy in chronic heart failure: a meta-analysis of 2,686 patients in 12 randomized trials. PLoS ONE. 2013;8(3):e58287. 23472172 10.1371/journal.pone.0058287 PMC3589263

[5] Brunner-La Rocca HP, Bektas S. Biomarker guided therapy in chronic heart failure. Card Fail Rev. 2015;1(2):96–101. 28785440 10.15420/cfr.2015.1.2.96 PMC5490943

[6] Nuñez J, De la Espriella R, Rossignol P, Voors AA, Mullens W, Metra M et al. Congestion in heart failure: a Circulating biomarker-based perspective. A review from the biomarkers working group of the heart failure Association, European society of cardiology. Eur J Heart Fail. 2022 ;24(10):1751-1766. 10.1002/ejhf.2664 36039656

[7] Ibrahim NE, Januzzi JL. Established and emerging roles of biomarkers in heart failure. Circ Res. 2018;123(5):614–29. 30355136 10.1161/CIRCRESAHA.118.312706

[8] Castiglione V, Aimo A, Vergaro G, Saccaro L, Passino C, Emdin M. Biomarkers for the diagnosis and management of heart failure. Heart Fail Rev. 2022;27(2):625–43. 33852110 10.1007/s10741-021-10105-w PMC8898236

[9] Barry-Loncq De Jong M, Allach Y, Abou Kamar S, Clephas PRD, Brunner-La Rocca H-P, Handoko ML et al. The association between serially measured Circulating biomarker patterns and pulmonary artery pressures measured by invasive hemodynamic monitoring. J Card Fail. 2025; S1071-9164(25)00236-2. 10.1016/j.cardfail.2025.04.014 40414277

[10] Brankovic M, Akkerhuis KM, Mouthaan H, Brugts JJ, Manintveld OC, van Ramshorst J, et al. Cardiometabolic biomarkers and their Temporal patterns predict poor outcome in chronic heart failure (Bio-SHiFT Study). J Clin Endocrinol Metab. 2018;103(11):3954–64. 30113647 10.1210/jc.2018-01241

[11] Field RJ, Adamson C, Jhund P, Lewsey J. Joint modelling of longitudinal processes and time-to-event outcomes in heart failure: systematic review and exemplar examining the relationship between serum Digoxin levels and mortality. BMC Med Res Methodol. 2023;23(1):94. 37076796 10.1186/s12874-023-01918-4 PMC10114381

[12] Greene SJ, Bauersachs J, Brugts JJ, Ezekowitz JA, Lam CSP, Lund LH, et al. Worsening heart failure: Nomenclature, Epidemiology, and future directions: JACC review topic of the week. J Am Coll Cardiol. 2023;81(4):413–24. 36697141 10.1016/j.jacc.2022.11.023

[13] Kennel PJ, Rosenblum H, Axsom KM, Alishetti S, Brener M, Horn E, et al. Remote cardiac monitoring in patients with heart failure: A review. JAMA Cardiol. 2022;7(5):556–64. 34964805 10.1001/jamacardio.2021.5090

[14] Ibrahim NE, Januzzi JL. Jr. Beyond natriuretic peptides for diagnosis and management of heart failure. Clin Chem. 2017;63(1):211–22. 28062619 10.1373/clinchem.2016.259564

[15] Morrow DA, de Lemos JA. Benchmarks for the assessment of novel cardiovascular biomarkers. Circulation. 2007;115(8):949–52. 17325253 10.1161/CIRCULATIONAHA.106.683110

[16] Kim SE, Cho DH, Kim JY, Kang SM, Cho MC, Lee HY, et al. Impact of NT-proBNP on prognosis of acute decompensated chronic heart failure versus de Novo heart failure. Int J Cardiol. 2022;363:163–70. 35753618 10.1016/j.ijcard.2022.06.055

[17] Bouwens E, Brankovic M, Mouthaan H, Baart S, Rizopoulos D, van Boven N et al. Temporal patterns of 14 blood biomarker candidates of cardiac remodeling in relation to prognosis of patients with chronic heart Failure-The Bio-SHiFT study. J Am Heart Assoc. 2019;8(4). 10.1161/JAHA.118.009555 PMC6405680 30760105

[18] Shimizu N, Kotani K. Point-of-care testing of (N-terminal pro) B-type natriuretic peptide for heart disease patients in home care and ambulatory care settings. Practical Lab Med. 2020;22. 10.1016/j.plabm.2020.e00183 PMC7585141 33134469

[19] Maisel A, Barnard D, Jaski B, Frivold G, Marais J, Azer M, et al. Primary results of the HABIT trial (Heart failure assessment with BNP in the Home). J Am Coll Cardiol. 2013;61(16):1726–35. 23500322 10.1016/j.jacc.2013.01.052

[20] McDonald K, Troughton R, Dahlström U, Dargie H, van der Krum H, et al. Daily home BNP monitoring in heart failure for prediction of impending clinical deterioration: results from the HOME HF study. Eur J Heart Fail. 2018;20(3):474–80. 29314505 10.1002/ejhf.1053

[21] Barry - Loncq de Jong M, Allach Y, Abou Kamar S, Clephas PRD, Brunner-La Rocca HP, Handoko ML et al. The association between serially measured Circulating biomarker patterns and pulmonary artery pressures measured by invasive hemodynamic monitoring. J Card Fail. 2025 23:S1071-9164(25)00236-2. 10.1016/j.cardfail.2025.04.014 40414277

[22] Núñez J, De la Espriella R, Rossignol P, Voors AA, Mullens W, Metra M, et al. Congestion in heart failure: a Circulating biomarker-based perspective. A review from the biomarkers working group of the heart failure Association, European society of cardiology. Eur J Heart Fail. 2022;24(10):1751–66. 36039656 10.1002/ejhf.2664

[23] Nassif ME, Nguyen DAN, Spertus JA, Gosch KL, Tang F, Windsor SL, et al. Association between change in ambulatory pulmonary artery pressures and natriuretic peptides in patients with heart failure: results from the EMBRACE-HF trial. J Card Fail. 2023;29(9):1324–8. 37230315 10.1016/j.cardfail.2023.05.009

[24] Piek A, Du W, de Boer RA, Sillje HHW. Novel heart failure biomarkers: why do we fail to exploit their potential? Crit Rev Clin Lab Sci. 2018;55(4):246–63. 29663841 10.1080/10408363.2018.1460576

[25] Voors AA, Kremer D, Geven C, ter Maaten JM, Struck J, Bergmann A, et al. Adrenomedullin in heart failure: pathophysiology and therapeutic application. Eur J Heart Fail. 2019;21(2):163–71. 30592365 10.1002/ejhf.1366 PMC6607488

[26] Kremer D, ter Maaten JM, Voors AA. Bio-adrenomedullin as a potential quick, reliable, and objective marker of congestion in heart failure. Eur J Heart Fail. 2018;20(9):1363–5. 29932477 10.1002/ejhf.1245

[27] Pandhi P, ter Maaten JM, Emmens JE, Struck J, Bergmann A, Cleland JG, et al. Clinical value of pre-discharge bio-adrenomedullin as a marker of residual congestion and high risk of heart failure hospital readmission. Eur J Heart Fail. 2020;22(4):683–91. 31797505 10.1002/ejhf.1693

[28] Kozhuharov N, Ng L, Wussler D, Strebel I, Sabti Z, Hartmann O, et al. Activity of the adrenomedullin system to personalise post-discharge diuretic treatment in acute heart failure. Clin Res Cardiol. 2022;111(6):627–37. 34302189 10.1007/s00392-021-01909-9 PMC9151518

[29] Obokata M, Kane GC, Reddy YNV, Melenovsky V, Olson TP, Jarolim P, Borlaug BA. The neurohormonal basis of pulmonary hypertension in heart failure with preserved ejection fraction. Eur Heart J. 2019;40(45):3707–17. 31513270 10.1093/eurheartj/ehz626 PMC7963136

[30] Allach Y, Brugts JJ. The role of serial cardiac biomarkers in prognostication and risk prediction of chronic heart failure: additional scientific insights with hemodynamic feedback. Expert Rev Cardiovas. 2023;21(2):97–109. 10.1080/14779072.2023.2177635 36744389

[31] DeLeon-Pennell KY, Meschiari CA, Jung M, Lindsey ML. Matrix metalloproteinases in myocardial infarction and heart failure. Prog Mol Biol Transl. 2017;147:75–100. 10.1016/bs.pmbts.2017.02.001 PMC5576003 28413032

[32] Sanchis L, Andrea R, Falces C, Llopis J, Morales-Ruiz M, López-Sobrino T, et al. Prognosis of new-onset heart failure outpatients and collagen biomarkers. Eur J Clin Invest. 2015;45(8):842–9. 26077878 10.1111/eci.12479

[33] Chang YY, Chen AR, Wu XM, Hsu TP, Liu LYD, Chen YH, et al. Comparison the prognostic value of Galectin-3 and serum markers of cardiac extracellular matrix turnover in patients with chronic systolic heart failure. Int J Med Sci. 2014;11(11):1098–106. 25170292 10.7150/ijms.8083 PMC4147635

[34] Abou Kamar S, Bracun V, El-Qendouci M, Bomer N, Bakker SJL, Gansevoort RT et al. Association of baseline and longitudinal changes in insulin-like growth factor-binding protein-7 with the risk of incident heart failure: data from the PREVEND study. Eur J Heart Fail. 2025 ;27(9):1755-17632024. 10.1002/ejhf.3328 PMC12502453 39015086

[35] Bracun V, van Essen B, Voors AA, van Veldhuisen DJ, Dickstein K, Zannad F et al. Insulin-like growth factor binding protein 7 (IGFBP7), a link between heart failure and senescence. Esc Heart Fail. 2022; 9(6):4167-4176. 10.1002/ehf2.14120 PMC9773704 36088651

[36] Nägele H, Bahlo M, Klapdor R, Schaeperkoetter D, Rödiger W. CA 125 and its relation to cardiac function. Am Heart J. 1999;137(6):1044–9. 10347329 10.1016/s0002-8703(99)70360-1

[37] Miñana G, de la Espriella R, Mollar A, Santas E, Núñez E, Valero E, et al. Factors associated with plasma antigen carbohydrate 125 and amino-terminal pro-B-type natriuretic peptide concentrations in acute heart failure. Eur Heart J-Acute Ca. 2020;9(5):437–47. 10.1177/2048872620908033 32129669

[38] Nunez J, de la Espriella R, Minana G, Santas E, Llacer P, Nunez E, et al. Antigen carbohydrate 125 as a biomarker in heart failure: a narrative review. Eur J Heart Fail. 2021;23(9):1445–57. 34241936 10.1002/ejhf.2295

[39] Núñez J, Núñez E, Bayés-Genís A, Fonarow GC, Miñana G, Bodí V, et al. Long-term serial kinetics of N-terminal pro B-type natriuretic peptide and carbohydrate antigen 125 for mortality risk prediction following acute heart failure. Eur Heart J-Acute Ca. 2017;6(8):685–96. 10.1177/2048872616649757 27199489

[40] Núñez J, Llàcer P, García-Blas S, Bonanad C, Ventura S, Núñez JM, et al. CA125-Guided diuretic treatment versus usual care in patients with acute heart failure and renal dysfunction. Am J Med. 2020;133(3):370–. 31422111 10.1016/j.amjmed.2019.07.041

[41] Chan MM, Santhanakrishnan R, Chong JP, Chen Z, Tai BC, Liew OW, et al. Growth differentiation factor 15 in heart failure with preserved vs. reduced ejection fraction. Eur J Heart Fail. 2016;18(1):81–8. 26497848 10.1002/ejhf.431

[42] Dahl CP, Husberg C, Gullestad L, Waehre A, Damås JK, Vinge LE et al. Increased Production of CXCL16 in Experimental and Clinical Heart Failure. Circulation: Heart Failure. 2009;2(6):624 – 32. 10.1161/CIRCHEARTFAILURE.108.821074 19919988

[43] Borst O, Schaub M, Walker B, Sauter M, Muenzer P, Gramlich M, et al. CXCL16 is a novel diagnostic marker and predictor of mortality in inflammatory cardiomyopathy and heart failure. Int J Cardiol. 2014;176(3):896–903. 25223819 10.1016/j.ijcard.2014.08.033

[44] Lehrke M, Millington SC, Lefterova M, Cumaranatunge RG, Szapary P, Wilensky R, et al. CXCL16 is a marker of inflammation, atherosclerosis, and acute coronary syndromes in humans. J Am Coll Cardiol. 2007;49(4):442–9. 17258089 10.1016/j.jacc.2006.09.034

[45] Velissaris D, Zareifopoulos N, Koniari I, Karamouzos V, Bousis D, Gerakaris A, et al. Soluble urokinase plasminogen activator receptor as a diagnostic and prognostic biomarker in cardiac disease. J Clin Med Res-Can. 2021;13(3):133–42. 10.14740/jocmr4459 PMC8016523 33854652

[46] Clark H, Palaniyar N, Strong P, Edmondson J, Hawgood S, Reid KBM. Surfactant protein D reduces alveolar macrophage apoptosis in Vivo1. J Immunol. 2002;169(6):2892–9. 12218102 10.4049/jimmunol.169.6.2892

[47] Brankovic M, Akkerhuis KM, Mouthaan H, Constantinescu A, Caliskan K, van Ramshorst J, et al. Utility of Temporal profiles of new cardio-renal and pulmonary candidate biomarkers in chronic heart failure. Int J Cardiol. 2019;276:157–65. 30098826 10.1016/j.ijcard.2018.08.001

[48] Gargiulo P, Banfi C, Ghilardi S, Magrì D, Giovannardi M, Bonomi A et al. Surfactant-Derived proteins as markers of alveolar membrane damage in heart failure. PLoS ONE. 2014; 9(12):e115030. 10.1371/journal.pone.0115030 10.1371/journal.pone.0115030 PMC4267772 25514679

[49] Bardin N, Moal V, Anfosso F, Daniel L, Brunet P, Sampol J, George FD. Soluble CD146, a novel endothelial marker, is increased in physiopathological settings linked to endothelial junctional alteration. Thromb Haemostasis. 2003;90(5):915–20. 14597988 10.1160/TH02-11-0285

[50] Gayat E, Caillard A, Laribi S, Mueller C, Sadoune M, Seronde MF, et al. Soluble CD146, a new endothelial biomarker of acutely decompensated heart failure. Int J Cardiol. 2015;199:241–7. 26209827 10.1016/j.ijcard.2015.07.039

[51] Malka K, Liaw L. NOTCH3 as a modulator of vascular disease : a target in Elastin deficiency and arterial pathologies. J Clin Invest. 2022;132(5):e157007. 10.1172/JCI157007 10.1172/JCI157007 PMC8884893 35229725

[52] Ferreira JP, Duarte K, Woehrle H, Cowie MR, Wegscheider K, Angermann C, et al. Biomarkers in patients with heart failure and central sleep apnoea: findings from the SERVE-HF trial. ESC Heart Fail. 2020;7(2):503–11. 31951323 10.1002/ehf2.12521 PMC7160494

[53] Tsutsui H, Albert NM, Coats AJS, Anker SD, Bayes-Genis A, Butler J, et al. Natriuretic peptides: role in the diagnosis and management of heart failure: A scientific statement from the heart failure association of the European society of Cardiology, heart failure society of America and Japanese heart failure society. Eur J Heart Fail. 2023;25(5):616–31. 37098791 10.1002/ejhf.2848

[54] McDonagh TA, Metra M, Adamo M, Gardner RS, Baumbach A, Bohm M, et al. 2021 ESC guidelines for the diagnosis and treatment of acute and chronic heart failure. Eur Heart J. 2021;42(36):3599–726. 34447992 10.1093/eurheartj/ehab368

[55] Horiuchi Y, Villacorta H, Maisel A. Natriuretic Peptide-guided therapy for heart failure. Heart Int. 2022;16:112. 36741100 10.17925/HI.2022.16.2.112 PMC9872778

[56] Pandhi P, Ter Maaten JM, Anker SD, Ng LL, Metra M, Samani NJ, et al. Pathophysiologic processes and novel biomarkers associated with congestion in heart failure. JACC Heart Fail. 2022;10(9):623–32. 36049813 10.1016/j.jchf.2022.05.013

[57] Bayes-Genis A, de Antonio M, Galan A, Sanz H, Urrutia A, Cabanes R, et al. Combined use of high-sensitivity ST2 and NTproBNP to improve the prediction of death in heart failure. Eur J Heart Fail. 2012;14(1):32–8. 22179033 10.1093/eurjhf/hfr156

[58] Gardner RS, Ozalp F, Murday AJ, Robb SD, McDonagh TA. N-terminal pro-brain natriuretic peptide - A new gold standard in predicting mortality in patients with advanced heart failure. Eur Heart J. 2003;24(19):1735–43. 14522568 10.1016/j.ehj.2003.07.005

[59] Zile MR, Claggett BL, Prescott MF, McMurray JJ, Packer M, Rouleau JL, et al. Prognostic implications of changes in N-Terminal Pro-B-Type natriuretic peptide in patients with heart failure. J Am Coll Cardiol. 2016;68(22):2425–36. 27908347 10.1016/j.jacc.2016.09.931

[60] Davarzani N, Sanders-van Wijk S, Karel J, Maeder MT, Leibundgut G, Gutmann M, et al. N-Terminal Pro-B-Type natriuretic Peptide-Guided therapy in chronic heart failure reduces repeated Hospitalizations-Results from TIME-CHF. J Card Fail. 2017;23(5):382–9. 28232046 10.1016/j.cardfail.2017.02.001

[61] Felker GM, Anstrom KJ, Adams KF, Ezekowitz JA, Fiuzat M, Houston-Miller N, et al. Effect of natriuretic Peptide-Guided therapy on hospitalization or cardiovascular mortality in High-Risk patients with heart failure and reduced ejection fraction A randomized clinical trial. Jama-J Am Med Assoc. 2017;318(8):713–20. 10.1001/jama.2017.10565 PMC5605776 28829876

[62] Stienen S, Salah K, Moons AH, Bakx AL, van Pol P, Kortz RAM, et al. NT-proBNP (N-Terminal pro-B-Type natriuretic Peptide)-Guided therapy in acute decompensated heart failure. Circulation. 2018;137(16):1671–83. 29242350 10.1161/CIRCULATIONAHA.117.029882

[63] Januzzi JL Jr., Rehman SU, Mohammed AA, Bhardwaj A, Barajas L, Barajas J, et al. Use of amino-terminal pro-B-type natriuretic peptide to guide outpatient therapy of patients with chronic left ventricular systolic dysfunction. J Am Coll Cardiol. 2011;58(18):1881–9. 22018299 10.1016/j.jacc.2011.03.072

[64] Pfisterer M, Buser P, Rickli H, Gutmann M, Erne P, Rickenbacher P, et al. BNP-Guided vs Symptom-Guided heart failure therapy the trial of intensified vs standard medical therapy in elderly patients with congestive heart failure (TIME-CHF) randomized trial. Jama-J Am Med Assoc. 2009;301(4):383–92. 10.1001/jama.2009.2 19176440

[65] Lainchbury JG, Troughton RW, Strangman KM, Frampton CM, Pilbrow A, Yandle TG, et al. N-terminal pro-B-type natriuretic peptide-guided treatment for chronic heart failure: results from the BATTLESCARRED (NT-proBNP-Assisted treatment to lessen serial cardiac readmissions and Death) trial. J Am Coll Cardiol. 2009;55(1):53–60. 20117364 10.1016/j.jacc.2009.02.095

[66] Albar Z, Albakri M, Hajjari J, Karnib M, Janus SE, Al-Kindi SG. Inflammatory markers and risk of heart failure with reduced to preserved ejection fraction. Am J Cardiol. 2022;167:68–75. 34986991 10.1016/j.amjcard.2021.11.045

[67] Scholte NTB, van Ravensberg AE, Shakoor A, Boersma E, Ronner E, de Boer RA et al. A scoping review on advancements in noninvasive wearable technology for heart failure management. Npj Digit Med. 2024;7(1):279. 10.1038/s41746-024-01268-5 10.1038/s41746-024-01268-5 PMC11470936 39396094

[68] Noci F, Capodici A, Nuti S, Passino C, Emdin M, Giannoni A. Wearable technologies to predict and prevent and heart failure hospitalizations: a systematic review. Eur Heart J - Digit Health. 2025;6(5):868–77. 40984981 10.1093/ehjdh/ztaf079 PMC12450522

[69] Devin J, Powell E, McGagh D, Jones T, Wang B, Le Page P et al. Non-Invasive wearable technology to predict heart failure decompensation. J Clin Med. 2025;14(20):7423. 10.3390/jcm14207423 10.3390/jcm14207423 PMC12565691 41156292

[70] Sun ZY, Wang ZH, Yun ZJ, Sun XN, Lin JG, Zhang XX, et al. Machine learning-based model for worsening heart failure risk in Chinese chronic heart failure patients. Esc Heart Fail. 2025;12(1):211–28. 39243185 10.1002/ehf2.15066 PMC11769658

[71] Bayes-Genis A, Pagnesi M, Codina P, Abraham WT, Amir O, de Boer RA, et al. Remote pulmonary artery pressure-guided management of patients with heart failure: A clinical consensus statement of the heart failure association (HFA) of the ESC. Eur J Heart Fail. 2025;27(9):1644–57. 40288763 10.1002/ejhf.3619 PMC12502462

[72] Brugts JJ, Radhoe SP, Clephas PRD, Aydin D, van Gent MWF, Szymanski MK et al. Remote haemodynamic monitoring of pulmonary artery pressures in patients with chronic heart failure (MONITOR-HF): a randomised clinical trial. Lancet. 2023;401(10394):2113-2123. 10.1016/S0140-6736(23)00923-6 37220768

[73] Perl L, Soifer E, Bartunek J, Erdheim D, Köhler F, Abraham WT, Meerkin D. A novel wireless left atrial pressure monitoring system for patients with heart Failure, first Ex-Vivo and animal experience. J Cardiovasc Transl. 2019;12(4):290–8. 10.1007/s12265-018-9856-3 30604310

[74] Uriel N, Bhatt K, Kahwash R, Mcminn TR, Patel MR, Lilly S, et al. Safety and feasibility of an implanted inferior Vena Cava sensor for accurate volume assessment: FUTURE-HF2 trial. J Card Fail. 2025;31(2):369–76. 39349159 10.1016/j.cardfail.2024.09.003

[75] Abraham WT, Adamson PB, Bourge RC, Aaron MF, Costanzo MR, Stevenson LW, et al. Wireless pulmonary artery haemodynamic monitoring in chronic heart failure: a randomised controlled trial. Lancet. 2011;377(9766):658–66. 21315441 10.1016/S0140-6736(11)60101-3

[76] Boehmer JP, Hariharan R, Devecchi FG, Smith AL, Molon G, Capucci A, et al. A multisensor algorithm predicts heart failure events in patients with implanted devices results from the multisense study. Jacc-Heart Fail. 2017;5(3):216–25. 28254128 10.1016/j.jchf.2016.12.011

[77] Gardner RS, Singh JP, Stancak B, Nair DG, Cao M, Schulze C et al. HeartLogic Multisensor Algorithm Identifies Patients During Periods of Significantly Increased Risk of Heart Failure Events Results From the MultiSENSE Study. Circ-Heart Fail. 2018;11(7):e004669. 10.1161/CIRCHEARTFAILURE.117.004669 10.1161/CIRCHEARTFAILURE.117.004669 30002113

[78] Loepez-Azor JC, Torre ND, Carmena MDGC, Perez PC, Munera C, Clement IM et al. Clinical utility of heartLogic, a multiparametric telemonitoring System, in heart failure. Cardiac Fail Rev. 2022; ;8:e13. 10.15420/cfr.2021.35 10.15420/cfr.2021.35 PMC9062709 35516795

[79] Ming WJ, Zhu YD, Jiang WJ, Zhang J, Liu JX, Wu L, Qin YL. Advanced point-of-care biomarker testing for the diagnosis of cardiovascular diseases. Sens Bio-Sens Res. 2025;47 : 100747. 10.1016/j.sbsr.2025.100747

[80] Adler ED, Voors AA, Klein L, Macheret F, Braun OO, Urey MA, et al. Improving risk prediction in heart failure using machine learning. Eur J Heart Fail. 2020;22(1):139–47. 31721391 10.1002/ejhf.1628

[81] Shimizu N, Kotani K. Point-of-care testing of (N-terminal pro) B-type natriuretic peptide for heart disease patients in home care and ambulatory care settings. Pract Lab Med. 2020;22:e00183. 33134469 10.1016/j.plabm.2020.e00183 PMC7585141

[82] Yun JE, Park JE, Park HY, Lee HY, Park DA. Comparative effectiveness of telemonitoring versus usual care for heart failure: A systematic review and Meta-analysis. J Card Fail. 2018;24(1):19–28. 28939459 10.1016/j.cardfail.2017.09.006

